# Thyroid Hormone, Thyroid Hormone Metabolites and Mast Cells: A Less Explored Issue

**DOI:** 10.3389/fncel.2019.00079

**Published:** 2019-03-29

**Authors:** Elisa Landucci, Annunziatina Laurino, Lorenzo Cinci, Manuela Gencarelli, Laura Raimondi

**Affiliations:** ^1^Section of Pharmacology, Department of Health Sciences, University of Florence, Florence, Italy; ^2^Section of Pharmacology, Department of Neurology, Psychology, Drug Sciences and Child Health, University of Florence, Florence, Italy

**Keywords:** mast cells, thyroid hormone, T3, histamine, 3-iodothryonamine, T1AM, 3-iodothryoacetic acid, TA1

## Abstract

Mast cells are primary players in immune and inflammatory diseases. In the brain, mast cells are located at the brain side of the blood brain barrier (BBB) exerting a crucial role in protecting the brain from xenobiotic invasion. Furthermore, recent advances in neuroscience indicate mast cells may play an important role in glial cell-neuron communication through the release of mediators, including histamine. Interestingly, brain mast cells contain not only 50% of the brain histamine but also hormones, proteases and lipids or amine mediators; and cell degranulation may be triggered by different stimuli activating membrane bound receptors including the four types of histaminergic receptors. Among hormones, mast cells can store thyroid hormone (T3) and express membrane-bound thyroid stimulating hormone receptors (TSHRs), thus suggesting from one side that thyroid function may affect mast cells function, from the other that mast cell degranulation may impact on thyroid function. In this respect, the research on hormones in mast cells is scarce. Recent pharmacological evidence indicates the existence of a non-genomic portion of the thyroid secretion including thyroid hormone metabolites. Among which the 3,5 diiodothyronine (3,5-T2), 3-iodothyroanamine (T1AM) and 3-iodothyroacetic acid (TA1) are the most studied. All these compounds are endogenously occurring and found to be increased in inflammatory-based diseases involving mast cells. T1AM and TA1 induce, as T3, neuroprotective effects and itch but also hyperalgesia in rodents with a mechanism largely unknown but mediated by the release of histamine. Due to the rapid onset of their effectiveness they may trigger histamine release from a cell where it is “ready-to-be released,” i.e., mast cells. Following a very thin path which passes through old experimental and clinical evidence, at the light of novel acquisitions on endogenous T3 metabolites, we aim to stimulate the attention on the possibility that mast cell histamine may be the connector of a novel (neuro) endocrine pathway linking the thyroid with mast cells.

## Background

Mast cells are ubiquitous cells of hematopoietic origin, disseminated in the periphery and in the nervous system. In the brain, mast cells are few in number, ideally located to the meninges and perivascular area on the brain side of the blood-brain barrier (BBB), a strategic localization to initiate the detoxification of xenobiotics and/or vascular and immunological effects. In most species studied, mast cells show a typical localization allowing an ideal interaction with neurons. The mast cell population of the brain is not static and it increases in numbers, distribution and activation in response to a variety of environmental stimuli that are associated with altered behaviors and physio-pathological conditions (Cirulli et al., 1998). Furthermore, emerging experimental evidence indicates that mast cells play pivotal roles in directing brain cell differentiation and plasticity including sexual maturation and behavior (Lenz et al., 2018) and in neuroprotection. In respect of this latter, stabilization of mast cells might represent a novel neuroprotective strategy during excitotoxicity (Ocak et al., 2018). The understanding of the mechanisms underlying the roles of mast cells in the brain is one of the challenges of the modern neurosciences. The hope is that the definition of such issues may help to comprehend how the mismatching of pro-inflammatory/anti-inflammatory factors levels, among which those derived from mast cells, may drive behaviors and neuron activation/survival possibly indicating novel drug targets. The relationship between mast cells and neurons is also reproduced in somatosensory neurons.

Irrespective of their tissue distribution, the characteristic feature of mast cells is the presence of cytoplasmic secretory granules which, upon activation, spread in the microenvironment lipid mediators, amines and hormones other than proteoglycans including heparin. Actually, the composition of mast cell granules shows some kind of heterogeneity depending on their tissue localization (Beil et al., 2000) and it looks that the presence of charged compounds in the granules is essential for the storage of other mediators (Grujic et al., 2013). As to say that there is a sort of chemical essential requisite for filling mast cell granules. Irrespective of this, mast cell granules always contain histamine, a charged amine which can be secreted together with the other mediators.

Sporadic evidence indicates that cells of the immune system, including mast cells can synthesize and store hormones among which are thyroid stimulating hormone (TSH) and the thyroid hormone T3 (Csaba and Pállinger, 2009a,b; Thangam et al., 2018). T3 levels were found increased following cell exposure to low histamine concentrations (Csaba and Pállinger, 2009a). Furthermore, evidence also indicates that mast cells express T3 receptors and that tissue mast cells population increased in hypothyroidism (Siebler et al., 2002), thus suggesting that the health or sick thyroid could condition mast cell hormone levels and/or that mast cells may represent an alternative source of packaged T3 locally deliverable (Thangam et al., 2018). Actually, even if evidence that T3 content decreased in peritoneal mast cells from hypothyroid rats (Csaba, 2014), it could further sustain this dual role of mast cells, though a definitive proof of this duality is lacking. In this respect, the progress in understanding the roles of T3 metabolites might open a new scenario.

## Mast Cells and the Thyroid

The potential relationship, if any, between the thyroid and mast cells is certainly complex and likely two-fold. There is evidence that T3 effects can be modulated by mast cells, and that mast cells can modulate thyroid function. This interaction is clinically relevant in autoimmune disorders such as rheumatoid arthritis or intestinal inflammatory diseases (Costela-Ruiz et al., 2018) or in autoimmune thyroiditis disease such as chronic urticaria, characterized by recurrent episodes of mast cell-driven wheal and flare-type skin reactions, as well as in Grave’s and Hashimoto disease (Ruggeri et al., 2013). In patients with chronic urticaria and angioedema, the presence of thyroid autoantibodies (Leznoff et al., 1983) and IgE antibodies against thyroid peroxidase were identified. By binding on the surface of mast cells these anti-TPO-IgE autoantibodies cause mast cell activation and degranulation, thus playing an active role in the pathogenesis of chronic urticarial (Altrichter et al., 2011). Mast cells also play a pivotal role in a complication associated to Grave’s disease. van Steensel et al. (2012) demonstrated that in patients with Grave’s ophthalmopathy, the number of mast cells in orbital tissue increased and these cells were capable to activate, through the PDGF pathway, the orbital fibroblast causing the onset of ophthalmopathy. These data suggest mast cells not only as regulators of fibroblast activation, but also as a possible therapeutic target (van Steensel et al., 2012). In fact, in a small cohort of patients, a little clinical improvement after treatment with cetirizine was observed (Lauer et al., 2008). Another interaction between thyroid and mast cells takes place in the bone and in particular, in bone remodeling and endochondral bone formation. The effect of thyroid on these processes are well known but the specific mechanism is partially elucidated. T3 exerts direct actions on chondrocyte growth and mast cells located in the bone marrow closed to epiphyseal plate are able to interact with osteoblasts and chondrocytes were found involved in processes that modify cartilage matrix and influence mineralization, further supporting the link between thyroid function and mast cells in bone metabolism. In fact, the number and the distribution of bone marrow-derived mast cells was found influenced by the thyroid status. Interestingly, bone marrow mast cells express thyroid hormone receptors with cytoplasmic localization which limits receptor trafficking towards the nuclei. Interestingly, such evidence indicates that mast cells represent a conjunction ring between T3 and bone remodeling and opens to non-genomic effects of T3 in bone metabolism and differentiation (Siebler et al., 2002). Data identifying an influence of mast cells on the thyroid support the bivalence of the relationship between thyroid and mast cells. Rocchi et al. (2007) demonstrated a specific role of mast cells on thyroid function in non-thyroidal illness (NTI). NTI are defined as changes of the hypothalamic—pituitary—thyroid axis in patients suffering from illnesses not primarily originating in the thyroid. These clinical conditions are characterized by: (a) decreased T3 levels; (b) decreased T4 due to a reduced binding capacity and/or affinity of serum carrier proteins for T4; and (c) inappropriately normal or low levels of TSH with respect to the decreased thyroid hormone levels (Faber et al., 1981; Kaptein et al., 1982; Docter et al., 1993; Wilcox et al., 1994). In a mouse model of NTI, a specific link between mast cells and bacterial infection-induced hypothyroidism was also found. This study identifies a role for mast cells as sensors capable of controlling the homeostatic responsiveness of the hypothalamus—pituitary—thyroid axis through the activation of specific crystallizing fragment (Fc) receptors (FCgR3). The lack of response in mast cells deficient mice confirmed the pivotal role of these cells (Rocchi et al., 2007).

This link might be stronger in case of heterogeneous diseases related to abnormal mast cell activation or increased tissue mast cell number, including cutaneous and/or their systemic presentations whose diagnosis is based on the monitoring of serum tryptase, elevated 24-h urinary histamine metabolite (methylhistamine). Among these is mastocytosis, a pathology characterized by increased tissue mast cell number. Among clinical manifestations of mastocytosis are dermatological symptoms, including itch and urticaria, and also neurological and psychiatric disorders (Georgin-Lavialle et al., 2016) mainly supported by increased histamine levels.

## Histamine and the Thyroid

Histamine is essentially a pro-inflammatory mediator and if a low-grade inflammation is considered to be beneficial, sustained inflammation may generate abnormal cell behaviors. Histamine has different fate and effects in neuronal and not neuronal tissues. In not neural tissues, histamine deriving from mast cells, basophiles or enterochromaffin-like cells is recognized as a charged amine which enters in the calculation of the diamine (polyamine) tissue levels and it is implicated in local inflammation, pain, itch and vasodilatation. In periphery it is preferentially scavenged by the diamine oxidase (DAO) producing hydrogen peroxide and promotes its effects activating mainly the for types of receptors. In the central nervous system, histamine is produced and released by histaminergic neurons and by mast cells (Li et al., 2018) and the discrimination of the two possible sources can be achieved using genetic models or stabilizing pharmacologically mast cells. In the central nervous system, histamine is not considered a polyamine, the DAO is absent, and the enzymes involved in its metabolism, include the histamine methyl-transferase and the type B monoamine oxidase (MAO-B). Brain histamine is retained as a signal of the cell-to-cell communication with particular respect to microglia and neurons and the modulation of the brain histaminergic system may be afforded by drugs promoting histamine release acting at receptors, working somehow as “neuromodulators” or by triggering mast cell degranulation. To note, mast cells possess receptors for aminergic mediators including the four types of histaminergic receptors, a condition which allows histamine to establish a sort of paracrine control on mast cells degranulation (Thangam et al., 2018). Thus, the role of mast cells, where histamine is ready-to-be released, may became relevant in generating high local histamine levels and immune-mediated inflammatory milieus (Fang et al., 2014).

Brain histamine (neuronal and not neuronal) is part of the mediators involved in the control of hypothalamic governed behaviors and it is also endowed of neuroprotective effects against excitotoxic damage (Kukko-Lukjanov et al., 2006). Furthermore, brain histamine is also part of the mechanisms of the neuroprotection offered by T3 metabolites (Cao et al., 2009; Laurino et al., 2018a,b). On the other hand, it is well assessed that histamine also controls the release of TSH (Roberts and Calcutt, 1983), a finding potentially linking the histaminergic system to the control of thyroid function. Actually, data regarding the effect of some histaminergic type 2 receptor antagonists on patient T3 serum levels seem to support, at least in part, the role of histamine in the control of TSH (Pasquali et al., 1981; Corinaldesi et al., 1987), thus suggesting a possible role of histamine in thyroxine regulation.

The T3 levels are critical regulators of the prenatal and neonatal development of the nervous system and of post-natal brain plasticity. A part of the well-known role of T3 on neurons, T3 is also a critical regulator of glial cell functions with not only genomic but also non-genomic mechanisms. Glial cells, as mast cells, express T3 transporters (Mori et al., 2015).

Histamine levels were found high during the embryonic brain development (Pearce and Schanberg, 1969) and Sabria et al. (1987) reported T3 as possible candidate for controlling brain mast cells number and, consequently, the levels of brain histamine during development. Till now there is no evidence that brain mast cells may contain T3 but there are evidence that T3 metabolites activate the histaminergic system in the brain as well as in periphery (Laurino et al., 2018a,b).

Upadhyaya et al. (1993) demonstrated that in L-thyroxine-treated rats, histamine levels were found increased in the hypothalamus, thalamus and cortex of the rats, and that there was a positive correlation between circulating T3 and T4 levels and histamine. Csaba and Pállinger (2009a) demonstrated that very low histamine concentrations, not active on pain and inflammation, increased T3 mast cell content.

Inflammatory-based diseases of the thyroid are named thyroiditis. Banovac and De Forteza (1992) demonstrated that mast cells degranulation plays a pivotal role in promoting the early stage of thyroiditis. More recently, experimental data from Visciano et al. (2015) demonstrated that mast cells histamine promotes a pro-tumorogenic effect on the thyroid and, in thyroid tumors and in experimental secondary hypothyroidism as, the mast cell population of the gland increased (Melander et al., 1971; Melillo et al., 2010), thus increasing histamine content of the gland. Furthermore, supporting the role of inflammation and autoimmune diseases in thyroid cancers, evidence indicate that mast cells play a pro-tumorogenic role in human thyroid cancer promoting neoangiogenesis and invasiveness (Melillo et al., 2010). Furthermore, mast cell number increased in thyroid cancer particularly in the follicular variant of papillary thyroid carcinoma, where their localization could represent a diagnostic marker of this kind of tumor (Proietti et al., 2011). Furthermore, assuming that mast cells of the healthy or sick thyroids behave as peritoneal mast cells, their role would become relevant in the economy of T3 (Melander et al., 1975).

Among thyroid diseases manifestations are skin symptoms among which are including chronic urticaria, alopecia and atopic dermatitis (Artantas et al., 2009). Interestingly, in all these complex diseases the brain-skin connection and the pivotal role of stress-induced mast cell degranulation has been recently reviewed (Shimoda et al., 2010; Alexopoulos and Chrousos, 2016). In this respect, the local release of T3 or of T3 metabolites might participate in the clinical symptoms of these kin diseases.

To note, T3 been detected in peritoneal mast cells, a finding suggesting that these mobile cells may deliver the hormone systemically. Furthermore, Csaba and Pállinger (2009a,b) demonstrated that secondary to TSH receptor activation, mast cells T3 content was found increased, without however indicating the mechanism. Unfortunately, the knowledge on the physio-pathological significance of T3 presence in peritoneal mast cells did not improve further from the evidence of Csaba and Pállinger (2009a,b). Thus, the possibility that mast cells may function as an “alternative” thyroid gland remains only a hypothesis.

## Thyroid Hormone Metabolites and Histamine

Recent experimental evidence suggest that T3 metabolism generates compounds that are not only able to reproduce similar but also opposite effects to those of T3 without activating nuclear receptors. T3 metabolisms is carried out by decarboxylase, deiodinases and MAOs which can work on T3 in sequence or alternatively producing three main families of derivatives namely thyronines, thyronamines, thyroacetic and thyropropionic acids at different degree of iodination. To note, the activity of MAO allows the transformation of thyronamines into thyroacetic acids while deiodinase activity removes iodide ions without transforming the chemical family. Despite of this, the synthetic pathway of these compounds is not fully elucidated yet.

Interestingly, several experimental and clinical evidence suggest that T3 metabolites may be part of the thyroid homeostasis and that they could be implicated in thyroid diseases. Among the main derivatives studied are the 3,5 diiodothyronine (3,5-T2), the 3-iodothyronamine (T1AM) and 3-iodothyroacetic acid (TA1; Scanlan et al., 2004; Chiellini et al., 2012; Galli et al., 2012; la Cour et al., 2018). All of them were found endogenously in rodents and in humans with a tissue distribution of T1AM and TA1 mirroring that of the T3 (Chiellini et al., 2012). Some studies reported an increased T1AM levels in pathological conditions including diabetes (Galli et al., 2012) and heart failure (la Cour et al., 2018). Interestingly enough, 3,5-T2 circulating levels were found increased in cirrhosis, in brain tumors and in patients with non-thyroidal illnesses. In these latter cohort of patients, 3,5-T2 circulating levels were found correlated with the onset of post-surgical atrial fibrillation episodes (Dietrich et al., 2015). At the level of the heart, Frascarelli et al. (2011) demonstrated that T1AM exerts cardioprotective effects in an isolated rat heart model of ischemia-reperfusion injury, as to say that thyroid function on the heart is complex involving T3 and T3 metabolites.

Also, pharmacological evidence indicate T1AM is a regulator of body temperature (Scanlan et al., 2004) of mice feeding (Manni et al., 2012), it promotes memory acquisition and retrieval (Manni et al., 2013; Bellusci et al., 2017), it is anti-amnestic (Laurino et al., 2017), induces hyperalgesia and also has the potential to behave as an anti-obesity drug (Assadi-Porter et al., 2018). T1AM has a short half-life being rapidly degraded to its oxidative metabolite, the 3-iodothyroacetic acid (TA1). Since the oxidative deamination is carried on by MAO activity, TA1 is considered part of the pharmacological profile of T1AM, being MAO ubiquitously expressed (Laurino et al., 2015a,b). As a fingerprint of both T1AM and TA1, pharmacological effects is the rapid onset of their rapid onset, within 15 min from administration. Furthermore, they are active at very low doses always showing inverted bell-shaped dose-effect curves.

Laurino et al. (2015a,b, 2018a,b) demonstrated that T1AM and TA1 behavioral effects were dependent on the activation of the brain or peripheral histaminergic system, with a mechanism which, however, remains to be clarified.

T1AM is a multi-target compound (Bräunig et al., 2018) able to interact at G-protein coupled receptors, including the trace amine associated receptors, and also ion channels but not with T3 receptors. However, if we accept the trace amine associated receptors, the affinity of T1AM for such targets is much lower than its *in vivo* potency, thus making unlikely the participation of such targets in T1AM *in vivo* effects. Notwithstanding this, all the behavioral effects of T1AM (and of TA1) including the pro-learning effect, hyperalgesia and the neuroprotection were abolished by anti-histaminergic drug treatment of mice including type 1 receptor antagonists, a strategy which however does not allow to recognize the source of histamine which consists of neuronal and mast cell derived histamine. Considering T3 metabolites can pass the BBB reproducing most of the effects described for histamine, the timing of their effects, the localization of brain mast cells at the BBB, the possibility that mast cells, other than histaminergic neurons, are among the targets of T3 metabolites become a plausible hypothesis. This source of histamine would also explain the bell-shaped curves observed following T1AM (and TA1) administration where a slow re-synthesis does not allow a fast refilling of the granules. Furthermore, the link between T3 metabolites and their possible degranulating effect on mast cells might be more stringent in the case of peripheral histamine-mediated effects. In fact, T3 supplementation is one among the cause of systemic itch (Reamy et al., 2011) and pruritus is one among the clinical symptoms of hyperthyroidism (Ward and Bernhard, 2005). Similarly, T3 metabolites induce itch (Laurino et al., 2015a,b) activating, histamine-dependent, pERK in the dorsal root ganglia. This pathway is considered selective for mast cell-derived histamine-induced itch sensation (Dong and Dong, 2018; Huang et al., 2018). Even if the definitive proof is lacking, T3 metabolites, by activating mast cells, might be the mediators of T3-induced itch. Furthermore, confirming that itch and pain sensation have some common neuronal pathways, T1AM and TA1 also induce histamine-dependent hyperalgesia to thermal stimuli (Manni et al., 2013), a condition typically activating mast cells (Zhang et al., 2012).

**Figure 1:**
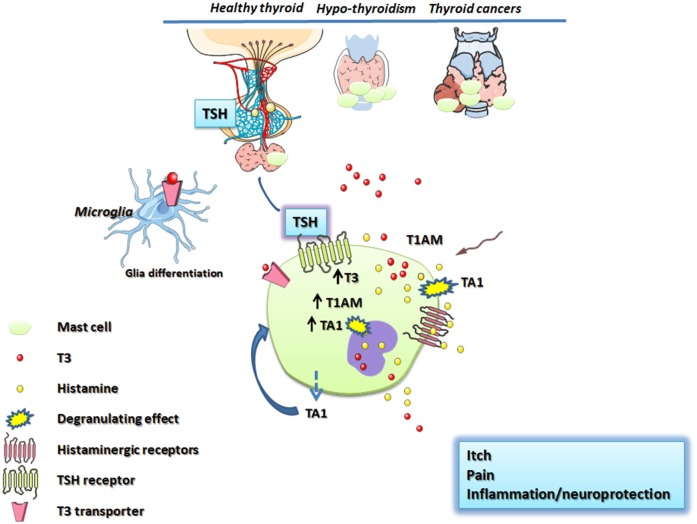
Schematic representation of thyroid and mast cell connections. The hypothalamus, throughout the release of the TSH, stimulates mast cells increasing the T3 content. T3 is co-stored with histamine in mast cell granules or is degraded to T1AM and/or TA1. T1AM and TA1 derived from circulation or produced inside mast cells trigger mast cell degranulation releasing T3 and histamine which mediates pain, itch and central effects including neuroprotection/neuroinflammation. Thyroid stimulating hormone (TSH); TSH receptor (TSHR); thyroid hormones (T3, T4); 3-iodothyronamine (T1AM); 3-iodothyroacetic acid (TA1); monoamine oxidases (MAO).

In conclusion, the relationship between the thyroid and mast cells is scarcely studied but we strongly believe it merits to be investigated further from the clinical and mechanistic point of view. In this respect in this article, we tried to point the attention on the non-canonical portion of the thyroid secretion constituted by T3 metabolites, as possible activators of mast cells and releaser of histamine (Figure 1).

## Author Contributions

All the authors participated in collecting and discussing the literature data.

## Conflict of Interest Statement

The authors declare that the research was conducted in the absence of any commercial or financial relationships that could be construed as a potential conflict of interest.
